# Cytocompatibility and Microbiological Effects of Ti6Al4V Particles Generated During Implantoplasty on Human Fibroblasts, Osteoblasts, and Multispecies Oral Biofilm

**DOI:** 10.3390/ma18245626

**Published:** 2025-12-15

**Authors:** Erika Vegas-Bustamante, Jorge Toledano-Serrabona, María Ángeles Sánchez-Garcés, Rui Figueiredo, Elena Demiquels-Punzano, Javier Gil, Luis M. Delgado, Gemma Sanmartí-García, Octavi Camps-Font

**Affiliations:** 1 School of Medicine and Health Sciences, Campus de Bellvitge, Universitat de Barcelona, C/Feixa Llarga s/n, Pavelló Govern 2ª Planta, Despatx 2.9, 08907 L’Hospitalet de Llobregat, Spain; erikavegasbustamante@ub.edu (E.V.-B.); masanchezg@ub.edu (M.Á.S.-G.); gemmasanmarti@ub.edu (G.S.-G.); ocamps@ub.edu (O.C.-F.); 2 Dental and Maxillofacial Pathology and Therapeutics Research Group, IDIBELL Research Institute, 08908 Barcelona, Spain; 3 Bioengineering Institute of Technology (BIT), Universitat International de Catalunya, 08017 Barcelona, Spain; elenademiquels@uic.es; 4 Bionspired Oral Biomaterials Interfaces, Departament de Ciencia e Ingenieria de Materiales, Escola d’Engingeria Barcelona Est, Universitat Politécnica de Catalunya, Av. Eduard Maristany 16, 08019 Barcelona, Spain; 5 Department of Graphic and Design Engineering, Universitat Politècnica de Catalunya (UPC), 08222 Terrasa, Spain

**Keywords:** dental implant, implantoplasty, fibroblast, osteoblast, Ti6Al4V

## Abstract

Objectives: This study aimed to evaluate the cytotoxic effects of Ti6Al4V particles and implantoplasty (IP)-treated surfaces on human fibroblasts and osteoblasts, and to investigate the influence of these particles on multispecies oral biofilm formation. Methods: Ti6Al4V particles generated during implantoplasty were collected. Human fibroblasts (HFF-1) and osteoblast-like cells (SaOs-2) were used to assess cytotoxicity through indirect lactate dehydrogenase (LDH) assays. Multispecies biofilms composed of *Streptococcus oralis*, *Actinomyces viscosus*, *Veillonella parvula* and *Porphyromonas gingivalis* were evaluated based on colony-forming units (CFUs) and metabolic activity. Fibroblasts and osteoblasts were co-cultured with biofilm-contaminated particles for 2, 4 and 6 h. Cell morphology and biofilm association were examined by phase-contrast microscopy, while metabolic activity was measured spectrophotometrically. Results: IP-treated surfaces showed no significant cytotoxicity (metabolic activity > 92%, LDH < 20%). Ti6Al4V particles selectively promoted *A. viscosus* and *V. parvula* growth (metabolic activity increases of ≈192% and ≈203%; CFU significantly higher versus controls, *p* < 0.05). Co-culture with biofilm-contaminated particles drastically reduced cell activity (fibroblasts < 25%, osteoblasts < 10%), whereas bacteria-free particles did not. Conclusions: Biofilm-contaminated particles released during implantoplasty markedly impair fibroblast and osteoblast cytocompatibility and selectively alter bacterial growth, whereas IP-treated surfaces per se are biocompatible. Minimizing particle dissemination and bacterial contamination during IP is therefore crucial.

## 1. Introduction

The peri-implant environment may be exposed to metallic particles generated by friction phenomena between the implant and the surrounding bone tissue, by micromovements at the implant–abutment interface, or as a result of aggressive decontamination procedures applied to the implant [1]. In many cases, these particles cannot be completely removed from the bone or peri-implant mucosal tissues, persisting within the surrounding tissue microenvironment.

Elevated concentrations of titanium (Ti) particles have been detected in dental implants affected by peri-implantitis [2,3], leading some authors to suggest a potential association between the presence of these particles and the development of the disease [4,5].

Such metal particles, particularly in nanoparticle form, can be internalized by various cell types in the peri-implant milieu, including macrophages and keratinocytes. Interaction with the cell membrane facilitates their uptake. In macrophages, this process may result in frustrated phagocytosis. Since the phagocytic process cannot be completed, macrophages might remain in an activated state, releasing pro-inflammatory cytokines, reactive oxygen species, and degradative enzymes. This sustained activation might promote a persistent inflammatory response that contributes to local tissue damage and osteoclast-mediated bone resorption. In fibroblasts, particle uptake occurs via endocytosis, which may impair cellular function. Once internalized, the particles in turn disrupt organelles and intracellular structures, leading to cellular dysfunction and damage [6,7].

Within this context, metal particles present in the peri-implant environment have been implicated in the activation of inflammatory pathways, promoting the secretion of proinflammatory cytokines such as tumor necrosis factor-alpha (TNF-α), interleukin-1 beta (IL-1β), and receptor activator of nuclear factor kappa-B ligand (RANKL) [8,9,10], and they may also downregulate osteogenic markers, including Runx2 and osteocalcin (OC) [11]. Additionally, titanium particles have been shown to stimulate fibroblasts to secrete proinflammatory cytokines, contributing to the chemotactic recruitment of monocytes/macrophages and the pathogenesis of aseptic loosening of implants [1,11]. These particles may further compromise the integrity of the oral epithelial barrier, disrupt cellular homeostasis, and induce DNA damage, as evidenced by the activation of genotoxic markers such as BRCA1 and CHK2 [8,12].

Implantoplasty (IP) is a mechanical decontamination technique aimed at removing bacterial biofilm and reducing surface roughness of the implant areas exposed to the oral cavity following resective or combined peri-implant surgical procedures [13,14,15,16,17,18,19,20]. This technique generates titanium debris and modifies the surface topography and physicochemical properties of the implant [1,21,22,23,24,25,26,27]. Once released, these particles are exposed to a complex oral environment where microbiological, biochemical and electrochemical factors—such as saliva, fluoride, and biofilm-derived metabolites—may exacerbate corrosion and degradation, accelerating deterioration of the metallic surface [1,28,29].

Fibroblasts and osteoblasts are critical for maintaining homeostasis in the peri-implant tissues, as they constitute the primary cellular components in these areas. A loss of cytocompatibility indicates potential impairment of these cells, thereby jeopardizing the balance and functionality of the peri-implant environment [12].

Although previous studies have shown that implantoplasty-induced surface modifications do not significantly affect the viability of fibroblasts [13,30] and osteoblasts [31], the Ti particles released during the procedure have been associated with a considerable decrease in the viability of both cell types [1,26,32].

While the cytotoxic potential of metallic debris has been examined, in more clinically relevant scenarios such as in the presence of a multispecies bacterial biofilm, its effect on fibroblasts and osteoblasts remains largely unexplored. Therefore, it is essential to characterize these particles in order to better understand their influence on cellular and biological responses.

The aim of this study was to evaluate the cytotoxic effects of metal particles and implant surfaces subjected to IP, both contaminated with a multispecies bacterial biofilm, by assessing cell viability and metabolic activity in human fibroblasts and osteoblasts. Additionally, biofilm-forming capacity in the presence of metal particles was investigated.

## 2. Materials and Methods

### 2.1. Preparation of Metallic Residues and Dental Implants

Metal particles generated during IP of 40 Titanium-6 Aluminum-4 Vanadium (Ti6Al4V) dental implants (Avinent Implant System S.L., Santpedor, Spain) were collected by the same researcher (E.V.B.). The surface of these implants was moderately rough (Sa ≈ 1.8 μm) as a result of sandblasting, acid etching, and anodizing techniques. The IP procedure was carried out following the simplified three-bur protocol described by Costa-Berenguer et al. [23], using a GENTLEsilence LUX 8000B turbine (KaVo Dental GmbH, Biberach an der Riß, Germany) under constant irrigation. The surface was sequentially modified using a fine-grained tungsten carbide bur (reference H379.314.014, KOMET GmbH & Co. KG, Lemgo, Germany), followed by a coarse-grained silicone polisher (reference 9608.314.030, KOMET GmbH & Co. KG, Lemgo, Germany), and finally a fine-grained silicone polisher (reference 9618.314.030, KOMET GmbH & Co. KG, Lemgo, Germany). A new set of burs and polishers was used for each implant. The samples were lyophilized to remove water from the metal released during IP. Additionally, 20 dental implants with a diameter of 4.5 mm and a length of 13 mm, featuring an internal hexagonal connection (Ocean E.C., Avinent Implant System S.L., Santpedor, Spain), underwent a 6-mm IP procedure following the same protocol. This protocol has been previously validated for its ability to standardize surface finishing after implantoplasty [33].

The specific surface area of the particles was measured using the Brunauer–Emmett–Teller (BET) theory under controlled vacuum conditions with nitrogen as the adsorbate. Particle size distribution was analyzed using a Mastersizer 3000 (Malvern Panalytical^®^, Malvern, UK) laser diffraction system, conducted in a wet medium with ethanol as the dispersing liquid. Mechanical and ultrasonic agitation were applied to prevent particle agglomeration. The morphology and chemical composition were examined using scanning electron microscopy (SEM) equipped with energy-dispersive X-ray spectroscopy (EDS) (Neon 40 Surface, Zeiss, Oberkochen, Germany).

### 2.2. Cell Culture

Two different cell lines purchased from the American Type Culture Collection (ATCC, Manassas, VA, USA) were used. Human foreskin fibroblasts (HFF-1) were cultured with Dulbecco’s Modified Eagle’s Medium (DMEM, Gibco, Waltham, MA, USA) and SaOs-2 with McCoy’s 5A medium (Gibco, Waltham, MA, USA). Both were supplemented with 15% Fetal Bovine Serum (FBS) and 1% Penicillin-Streptomycin (P/S) at 37 °C.

### 2.3. Cytotoxicity Evaluation

#### 2.3.1. Indirect Assay

The cytotoxicity of the sample was evaluated via indirect exposure determination according to the ISO 10993-5 standard [34]. Cytotoxicity tests were performed in triplicate. The samples studied were: Test sample: implants; Negative control: tissue culture plastic (TCP); Positive control: Triton.

For the indirect assay, 10 implants with IP were used and incubated at 37 °C with 9 mL of DMEM and McCoy’s 5A medium for three days. After this period, different dilutions were made with each medium (1:1, 1:5, 1:10, 1:50), reaching a final volume of 3 mL. Then, 5 × 10^3^ cells per well were seeded onto a 96-well plate and cultured for one day. The cells were then exposed to the different dilutions for up to 7 days. Cell response was evaluated in terms of metabolic activity at day 7 and cytotoxicity was assessed at days 1, 3 and 7, using TCP as negative (Control−) and Triton as positive (Control+) controls.

For metabolic activity, on day 7, a volume of 100 mL of resazurin (15 mg/mL) was added to each well, followed by incubation at 37 °C for 30 min. Then, media were transferred to a black 96-well plate to measure fluorescence in the spectrophotometer (Infinite M nano+, TECAN, Männedorf, Switzerland) at 560 nm excitation wavelength and 590 nm emission wavelength.

In relation to cellular viability, supernatants from days 1, 3 and 7 were kept at −20 °C and then lactate dehydrogenase (LDH) released from dead cells was quantified using a CyQUANT^TM^ LDH Cytotoxicity Assay kit (Thermo Fisher Scientific Inc., Waltham, MA, USA) following the manufacturer’s instructions. Briefly, 50 mL of the sample and 50 mL of the reactive solution were incubated at room temperature for 30 min in a 96-well plate. Then, absorbance was measured at 490 nm in the spectrophotometer (Infinite M nano+, TECAN).

Toxicity criteria were established according to the following ranges: 0–20%: non-toxic; 20–40%: slightly toxic; 40–60%: moderately toxic; >60%: Toxic [34].

#### 2.3.2. Direct Assay

For the direct assay, a cell concentration of 5 × 10^3^ cells per well was seeded with 10 implants with IP onto a low-adhesion 24-well plate and incubated for 7 days. Then, the implants were transferred to another 24-well plate and 1 mL of resazurin (15 mg/mL) was added and incubated at 37 °C for 30 min. Media were transferred to a black 96-well plate to measure fluorescence in the spectrophotometer (Infinite M nano+, TECAN) at 560 nm excitation wavelength and 590 nm emission wavelength. TCP was used as a negative control.

In the indirect assay (Section 2.3.1), cells were exposed only to culture media previously conditioned by implants subjected to implantoplasty, without any physical contact with particles. In the direct assay (Section 2.3.2), whole implants with implantoplasty were placed in direct contact with the cells.

### 2.4. Biofilm Formation with Particle Presence

#### 2.4.1. Biofilm Formation

Biofilm formation was adapted from a previous study [35], adjusting bacterial strains initially grown individually overnight with brain–heart infusion media (BHI, Oxoid concentrations). Four bacterial strains were used to develop a multispecies oral biofilm at 37 °C under anaerobic conditions (GasPak Anaerobic System, Becton Dickinson, Franklin Lakes, NJ, USA). The selected species represent initial colonizers, such as *Streptococcus oralis* (*S. oralis*, ATCC 6249) and *Actinomyces viscosus* (*A. viscosus*, ATCC 15987), early colonizers in the form of *Veillonella parvula* (*V. parvula*, ATCC 10790), and late colonizers such as *Porphyromonas gingivalis* (*P. gingivalis,* ATCC 33277). After incubation, the bacteria suspension was vortex for 5 s to disperse bacterial aggregations, and each bacterial strain was adjusted to the desired concentration. The concentration for individual bacteria studies was 8 × 10^7^ CFU/mL for all strains, while multispecies biofilms were developed with 1.6 × 10^7^ CFU/mL for *Actinomyces viscosus* and *Streptococcus oralis*, 4 × 10^7^ CFU/mL for *Veillonella parvula*, and 8 × 10^7^ CFU/mL for *Porphyromonas gingivalis*. Then, titanium particles, previously disinfected with 70% ethanol for 30 min and washed three times with phosphate-buffered saline (PBS) were placed in falcon tubes and incubated with inoculums. Subsequently, samples were incubated at 37 °C and under anaerobic conditions for 5 days. TCP was used as control.

Different initial CFU amounts were selected for each species based on their intrinsic growth rates and oxygen requirements, as described in a previous study [35].

#### 2.4.2. Biofilm Quantification

Biofilm quantification was performed in terms of metabolic activity and colony forming units (CFUs). For metabolic activity, media were removed and resazurin (15 mg/mL) was placed for 10 min. Then, fluorescence was quantified with a spectrophotometer (Infinite M Nano+, TECAN) at 560 nm excitation wavelength and 590 nm emission wavelength. Metabolic activity was presented as a reduction in resazurin, considering the metabolic activity of *S. oralis* with TCP as 100%.

Regarding CFU quantification, media were removed, and the content was resuspended with 500 mL of PBS, transferred to Eppendorf tubes and vigorously vortexed for 10 min. Serial dilutions were prepared for each sample and then placed on sterile media agar plates for 24 h. CFUs were manually counted.

### 2.5. Cell-Bacteria Co-Cultures

Two different co-cultures were performed to study fibroblasts versus multispecies biofilm and osteoblasts versus multispecies biofilm.

Human foreskin fibroblasts (ATCC) and SaOs-2 osteoblast cell line were culture in Dulbecco’s modified Eagle medium (DMEM) and McCoy’s 5a medium, supplemented with 10% and 15% fetal bovine serum (FBS), respectively. Cells were grown at 37 °C under 5% CO_2_ and 95% humidified air.

Fibroblasts and osteoblasts were seeded onto 24-well plates at 15 × 10^3^ cells/cm^2^. Co-cultures were performed under conditions adapted to the eukaryotic cells. The particles infected with the multispecies biofilm were incubated for 5 days, as previously described.

To achieve this, the bacterial suspension was centrifuged at 1000 rpm for 3 min and then resuspended in medium for fibroblast and osteoblast cells. A volume of 200 µL of concentrated infected particles were placed in each well. Co-cultures were incubated at 37 °C under 5% CO_2_ and 95% humidified air for 2, 4 and 6 h.

Phase contrast microscopy was used to monitor the co-cultures using an inverted microscope (Olympus CKX41 microscope with a Nikon DS-Fi1 camera, Tokyo, Japan). Metabolic activity was measured as previously explained; however, all samples were previously treated with 3× penicillin-streptomycin to minimize the presence of extracellular bacteria.

Co-cultures were performed in the presence of dental implants subjected to IP, referred to as the implant group. Two controls were used: TCP and control. Infected particles were cultured only with tissue culture plastic (referred to as TCP), and multispecies biofilm were incubated without particles in tissue culture plastic (referred to as control). In addition, all conditions were replicated without bacterial contamination to monitor fibroblast and osteoblast behavior, labeled as “wo” (without bacteria).

### 2.6. Statistical Analysis

The data obtained were entered into a Microsoft Excel spreadsheet (Microsoft^®^, Redmond, WA, USA) and subsequently processed using the Stata 14 statistical package (StataCorp^®^, College Station, TX, USA). To evaluate cytotoxicity through direct and indirect assays, the Kruskal–Wallis test followed by Dunn’s multiple comparison test was used, employing Prism 10 software.

## 3. Results

**Figure 1:**
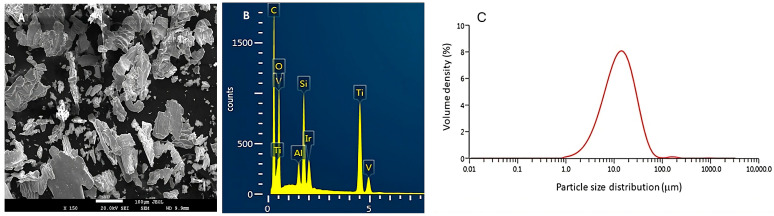
(**A**) SEM image of the metal particles of a Ti6Al4V sample after implantoplasty treatment. (**B**) EDS microanalysis spectrum of Ti6Al4V particles obtained after implantoplasty. (**C**) Particle size distribution of Ti6Al4V particles obtained after implantoplasty, showing a normal distribution (bell-shaped Gaussian curve).

The particles exhibited a predominantly planar morphology, consistent with the characteristics expected from the mechanical machining procedures employed. Figure 1A shows SEM images of the metal particles.

Energy-dispersive X-ray spectroscopy (EDS) analysis confirmed that the particles were composed of Ti6Al4V, with detectable residues of Si, C and oxygen originating from the burs used during the machining process (Figure 1B). The high carbon peak is expected due to the carbon coating applied to improve sample conductivity during SEM observation, as well as possible residues from silicon carbide burs used in implantoplasty. The apparent overlap of Ti, V, and O in the low-energy region occurs because their characteristic X-ray lines (Ti-L, V-L, and O-K) have very similar energies, resulting in peaks that appear nearly superimposed. Additionally, both Ti and V exhibit multiple characteristic lines (L and K series), which explains the presence of additional peaks at higher energies. This pattern is normal and expected in EDS analyses of Ti6Al4V alloys. As can be seen in Figure 1A, the particles show significant plastic deformation with ductile fractures and the presence of slip bands. With this morphology, it can be stated that the particles will have a very high surface energy and will be more susceptible to electrochemical corrosion processes in the body.

Particle size analysis revealed a range from 2–92 µm, following a normal distribution (Figure 1C). The average equivalent diameter was approximately 39 µm, and the size distribution curve indicated that 90% of the particles fell within the range of 25–70 µm. Additionally, the average specific surface area of the Ti6Al4V particles was 0.4532 ± 0.0987 m^2^/g.

### 3.1. Cytotoxicity Evaluation

The toxicity of the implants with implantoplasty was initially evaluated using indirect cell cultures with fibroblasts (HFF-1) and osteoblasts (SaOs-2). For this purpose, implants were incubated in culture media, and serial dilutions of the extracted media were exposed to the cells. This approach allows assessment only of compounds that leach out; however, it does not provide information on direct cell–surface interactions.

**Figure 2:**
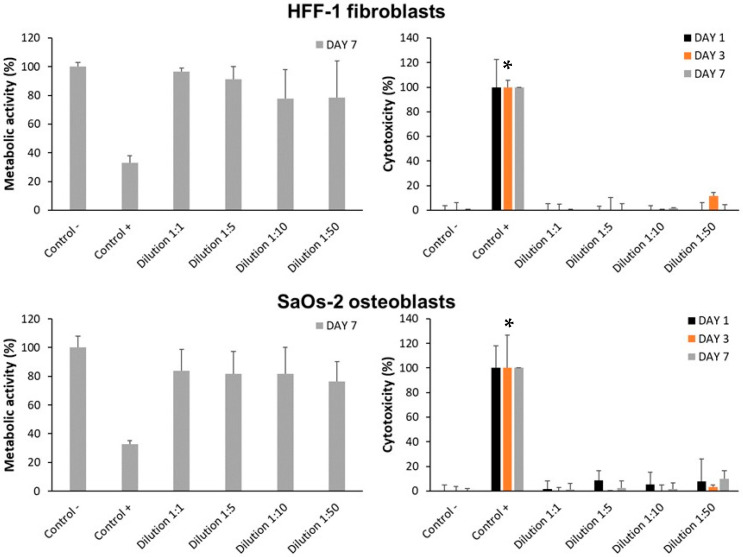
Indirect cell culture of HFF-1 fibroblasts and SaOs-2 osteoblasts exposed to extracts from dental implants subjected to implantoplasty. * *p* < 0.05.

Figure 2 presents the cytotoxicity measured on days 1, 3 and 7, as well as the metabolic activity on day 7, across different experimental groups.

The negative controls exhibited the highest metabolic activity (100%), indicating optimal cell viability. In both cell lines, neither the concentrated extract nor its serial dilutions significantly affected metabolic activity or induced cytotoxicity (<20%), compared to the negative controls (*p* > 0.05). The 1:50 dilution showed a slight increase in cytotoxicity in fibroblasts on day 3 (11.54%), though still within the non-toxic range. In contrast, the positive cytotoxic controls (Control+) showed markedly reduced metabolic activity and increased cytotoxicity (*p* = 0.0404; significant vs. Control−).

**Figure 3:**
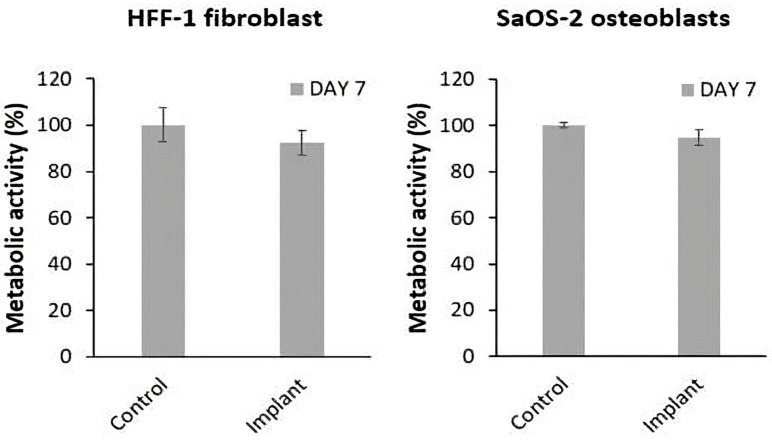
Direct cell culture of HFF-1 fibroblasts and SaOs-2 osteoblasts exposed to dental implants with implantoplasty. Control: TCP.

As toxicity due to leached compound was discarded, a direct cell culture was used to assess whether the cells actually interact with the particles. A similar trend was observed in direct cell cultures of fibroblasts and osteoblasts exposed to implants with implantoplasty (Figure 3). Under all conditions, metabolic activity remained at levels comparable to TCP (*p* = 0.3687). Specifically, the implant group showed 92.35% metabolic activity in fibroblasts and 94.78% in osteoblasts, compared to 100% in the control group.

### 3.2. Biofilm Formation with Particle Presence

**Figure 4:**
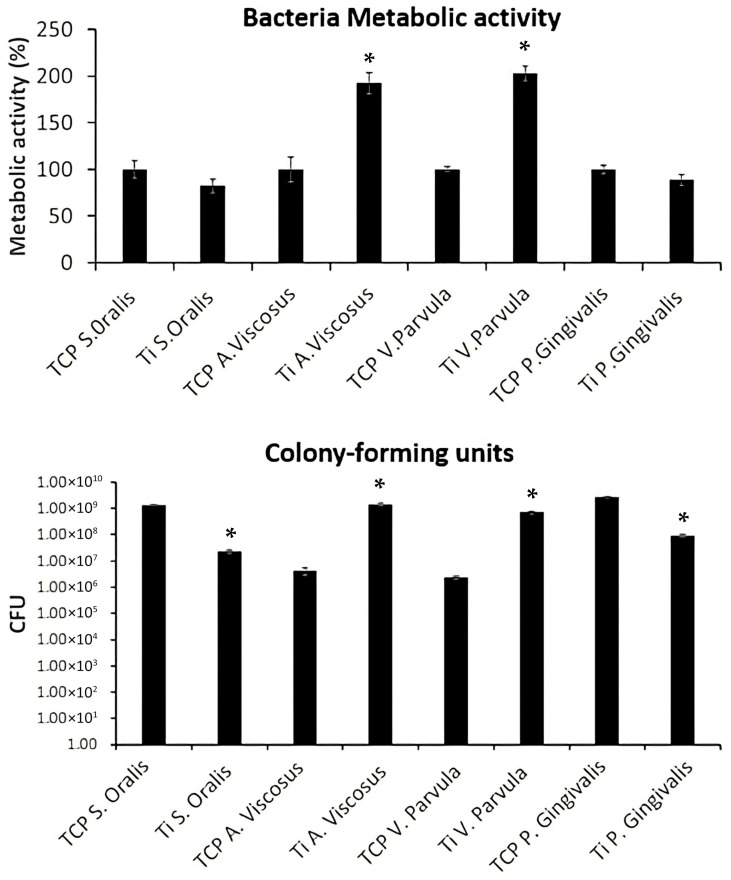
Development of single bacterial strain biofilms exposed to IP particles (Ti groups) measured in terms of metabolic activity and CFU quantification. TCP: tissue culture plastic with a single bacterial species; Ti: titanium particles with a single bacterial species. * *p* < 0.05 versus TCP.

Following the evaluation of the biological response of implants subject to implantoplasty, the effects of the metal particles were assessed on single-strain biofilms of *A. viscosus, S. oralis, V. parvula* and *P. gingivalis* (Figure 4). The development of biofilm exposed to IP particles was compared to TCP without traces of these particles. Interestingly, *A. viscosus* and *V. parvula* demonstrated a significant increase in metabolic activity, reaching 192.36% and 202.89%, respectively, compared to the TCP counterparts (*p* = 0.0152; significant vs. TCP). These findings were further confirmed by CFU quantification, where *A. viscosus* and *V. parvula* also showed higher CFU counts when cultured with these particles.

In contrast, *S. oralis* and *P. gingivalis* presented lower CFU counts (*p* = 0.0153; significant vs. TCP); however, their metabolic activity was comparable to that of the TCP controls (*p* = 0.1843). Therefore, *A. viscosus and V. parvula* proliferated significantly more in the presence of Ti.

**Figure 5:**
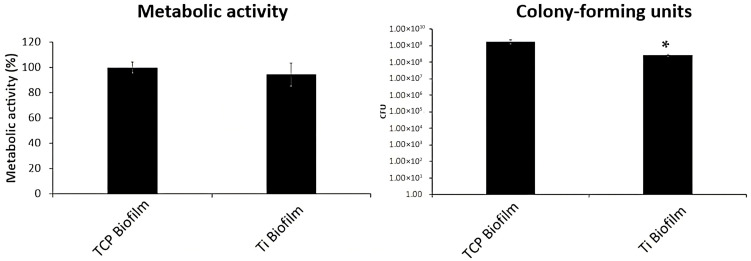
Development of multispecies biofilms exposed to IP particles (Ti groups) measured in terms of metabolic activity and CFU quantification. group Ti biofilms: titanium particles with biofilms; group TCP biofilms: tissue culture plastic with biofilms. * *p* < 0.05 versus TCP.

The analysis of the development of multispecies biofilm (Figure 5) revealed that biofilm metabolic activity was not affected by the presence of IP particles (*p* = 0.1843), specifically showing figures of 94.34% in the Ti biofilms group versus 100% in the TCP biofilms group.

However, CFU quantification demonstrated a slight reduction, similar to the trend observed for certain strains in the development of single-species biofilms. Specifically, the Ti biofilm group showed a count of 2.57 × 10^8^ CFUs, whereas the TCP biofilm group reached 1.75 × 10^9^ CFUs. This indicates that the number of CFUs in the Ti biofilm group was lower compared to the TCP group (*p* = 0.0152; significant vs. TCP).

### 3.3. Cell-Bacteria Co-Culture

**Figure 6:**
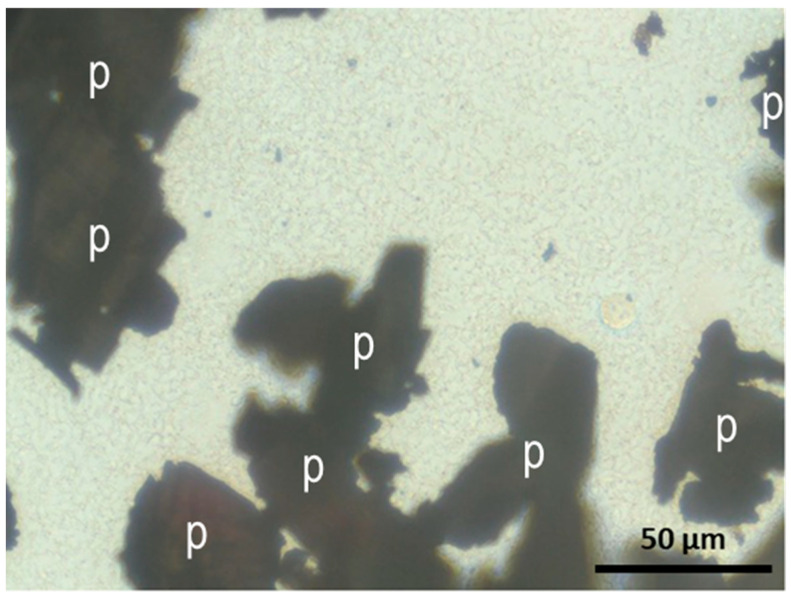
Planktonic bacteria released from the multispecies biofilm developed on IP particles (labeled with p) after two hours of culture.

Finally, an evaluation was conducted of the effect of contaminated particles with multispecies biofilm on fibroblast and osteoblast adhesion. As shown in Figure 6, bacteria from the multispecies biofilm on IP particles were released after two hours of culture, completely covering the microscope’s visual field. The co-culture period (2–6 h) was intentionally limited to capture early cellular responses, since longer incubation times would lead to excessive bacterial overgrowth that could mask these initial interactions and compromise reproducibility.

**Figure 7:**
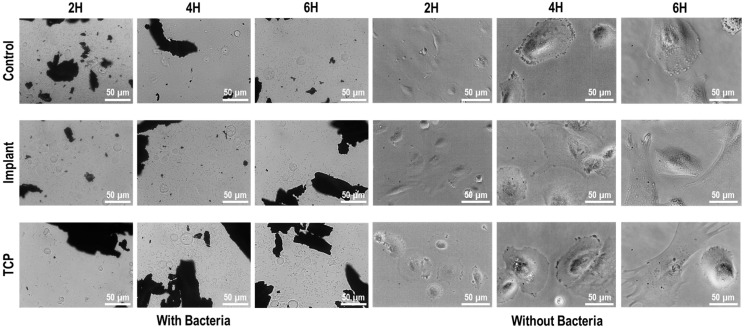
Fibroblast direct co-culture with multispecies biofilm-contaminated IP particles. TCP: tissue culture plastic with particles, both with and without biofilms; Implant: implant surface together with titanium particles, both with and without biofilms; Control: tissue culture plastic without particles, with and without biofilms.

**Figure 8:**
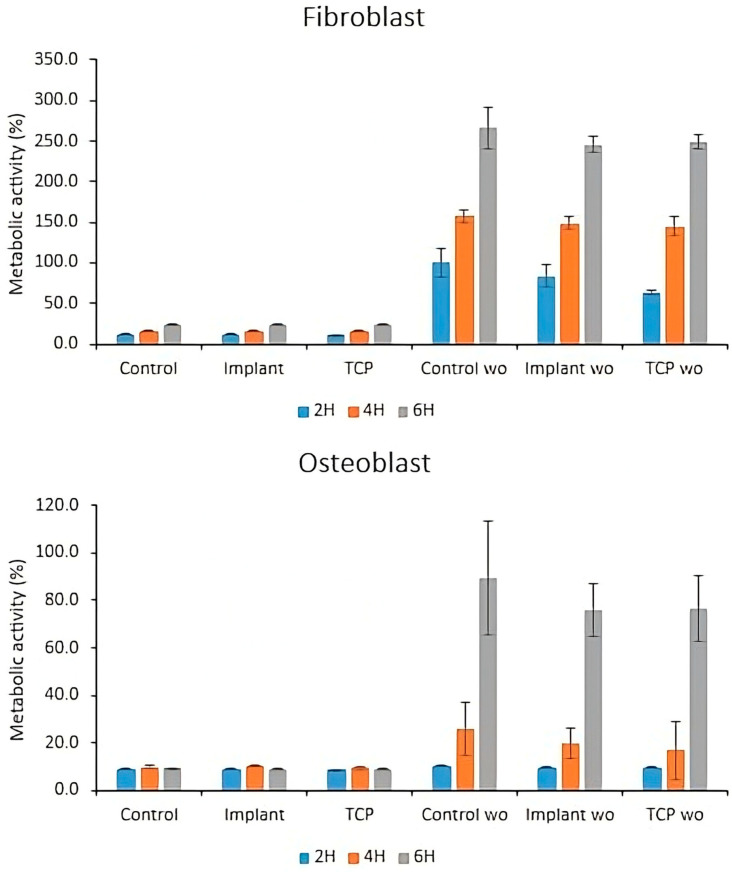
Metabolic activity of fibroblasts and osteoblasts directly cultured with and without bacteria. Control: tissue culture plastic with biofilms; Control Wo: without biofilms; Implant: implant surface together with titanium particles, with biofilms; Implant Wo: without biofilms; TCP: tissue culture plastic with particles and biofilms; TCP Wo: without biofilms.

When fibroblasts were cultured without bacterial contamination (Figure 7), cell adhesion was observed after as early as two hours, with signs of cell spreading and elongated morphology. By four hours, most cells exhibited an egg-shaped morphology with increased surface area, and spreading was largely complete after 6 h. In contrast, fibroblast adhesion was significantly impaired in the presence of bacterial contamination, as the cells retained a rounded morphology at all timepoints. These observations were confirmed by the metabolic activity measurements (Figure 8).

Metabolic activity values were calculated using the 2-h time point as the reference baseline. This approach can lead to values above 100%, since fibroblast activity and proliferation might increase after the above-mentioned time point. Thus, in this particular study, the observed high values in the bacteria-free groups probably reflect the expected early kinetics of resazurin reduction when the reference time point is set when cellular metabolic activity is still minimal.

**Figure 9:**
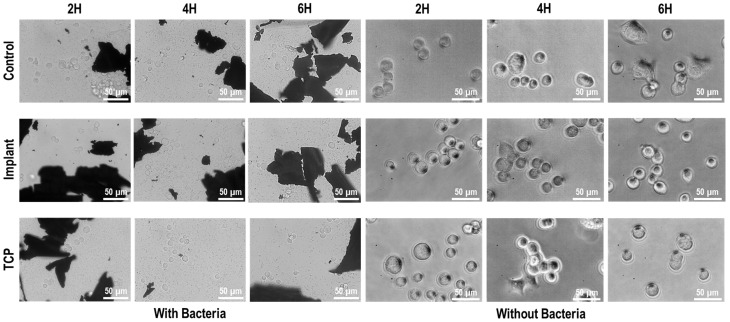
Osteoblast direct co-culture with multispecies biofilm-contaminated IP particles. (TCP): Tissue culture plastic with particles, both with and without biofilms; (Implant): Implant surface together with titanium particle, both with and without biofilms; (Control): Tissue culture plastic without particles, with and without biofilms.

As shown in Figure 7 and Figure 9, the metal particles appear less clearly defined in the ‘Without bacteria’ groups because the focal plane was adjusted to visualize cell morphology, which reduces the definition of dense materials under phase-contrast microscopy.

Similarly, when osteoblasts were cultured without bacterial contamination (Figure 9), the cells initially maintained a rounded morphology at two hours, with some adopting a polygonal shape after four hours and showing a slight increase in cell area after 6 h. In contrast, osteoblasts cultured with bacteria retained a rounded morphology at all timepoints, indicating complete inhibition of cell spreading. These findings were also corroborated by the metabolic activity measurements (Figure 8).

Figure 8 shows the metabolic activity (%) of fibroblasts and osteoblasts, measured at 2, 4 and 6 h (2H, 4H, 6H) in different groups: Control, Implant, TCP (all in the presence of bacteria) and the corresponding bacteria-free groups (wo).

The groups with bacteria showed low metabolic activity in fibroblasts (<25%), indicating a negative effect on their metabolism. In contrast, the groups without bacteria (“wo”) exhibited significantly higher metabolic activity, reaching levels above 245% at 6 h. This suggests that the absence of bacteria favors fibroblast proliferation and functionality. Osteoblasts exposed to bacteria showed very low metabolic activity (−10%), indicating strong inhibition of their function and survival. In contrast, without bacteria, metabolic activity increased significantly, reaching between 70% and 90% at 6 h. This suggests that the presence of bacteria strongly inhibits osteoblast metabolic activity, affecting their normal function and survival.

## 4. Discussion

The present study evaluated the cytotoxicity of metal particles released from both polished titanium implants and the implant surfaces subjected to implantoplasty (IP), all contaminated with a multispecies biofilm, under clinically relevant in vitro conditions. Cell viability was assessed in human fibroblasts and osteoblasts, and bacterial adhesion to these particles was quantified. The results demonstrated that titanium particles exposed to bacterial biofilm significantly reduced the cytocompatibility of both fibroblastic (Figure 7 and Figure 8) and osteoblastic cells (Figure 8 and Figure 9), as indicated by a marked decrease in their metabolic activity. In contrast, implant surfaces treated with IP in the absence of bacterial contamination showed no cytotoxic effects, with fibroblasts and osteoblasts maintaining metabolic activity above 92% and cytotoxicity levels below 20% (Figure 2 and Figure 3). These findings are particularly relevant from a clinical perspective, as they support the biocompatibility of IP-treated surfaces, which are commonly involved in the surgical treatment of peri-implantitis. This differential biological response between implant surfaces and detached metal particles highlights the importance of controlling particle dissemination during the procedure.

To the best of our knowledge, no previous studies have examined the combined impact of bacterial biofilms and titanium particles on fibroblasts and osteoblasts under such physiologically relevant in vitro conditions. Nevertheless, certain limitations should be acknowledged. Firstly, the study employed a single implant material, Ti6Al4V, whereas dental implants can also be manufactured from commercially pure titanium (cpTi) or alternative alloys such as TiZr, which may exhibit distinct ion-release profiles and cytotoxic responses. Therefore, future studies should also evaluate cpTi and other clinically relevant alloys to determine whether their degradation products elicit differential biological effects. Secondly, a specific milling protocol was used for implantoplasty, although multiple protocols have been described in the literature, potentially influencing both the amount and the composition of the generated debris. Finally, the short cell–bacteria co-culture period (2–6 h) enabled analysis of early cytotoxic responses but did not permit evaluation of longer-term effects such as apoptosis, inflammatory signaling, oxidative stress responses, or extracellular matrix production. Future work employing extended or dynamic co-culture systems is required to characterize these delayed cellular events.

In the oral cavity, corrosive substances such as fluorides, lactic acid, citric acid, and chlorides, present in saliva and oral biofilms, can induce corrosion of titanium surfaces, promoting the release of metal ions and particles [28,36]. Previous investigations into biofilm formation on titanium surfaces have underscored the crucial role of bacterial colonization in peri-implant diseases [35,37,38,39]. Surface roughness has been positively correlated with increased bacterial adhesion [35,37,40], supporting the importance of minimizing bacterial attachment in order to maintain peri-implant health.

In the present study, we intentionally excluded saliva, proteins, and host-derived mediators to isolate the specific contribution of multispecies biofilm–titanium interactions. Our aim was to analyze the early microbiologically induced degradation of Ti6Al4V under controlled conditions, minimizing additional variables with a direct effect on the biofilm. However, the use of saliva, combined with pH cycling and enzymatic activity, could have provided a more realistic representation of the oral environment. Thus, future studies should assess and compare the debris generated by the various materials and alloys used in implantology, incorporating more representative experimental parameters.

Multispecies oral biofilm models have been developed to replicate in vivo colonization, demonstrating strong affinity for titanium even when antibacterial coatings are present [37,41,42]. However, Godoy et al. [39] reported reduced adhesion of *S. sanguinis* and *L. salivarius* on titanium surfaces treated with antibacterial agents such as TESPSA, without inducing cytotoxic effects.

Despite these efforts, controversy persists regarding the way in which surface characteristics influence biofilm development and the progression of peri-implant disease [43,44]. Dysbiotic biofilms can initiate an inflammatory response that stimulates osteoclast activity and bone resorption [45]. Anaerobic conditions beneath the gingival margin further favor the proliferation of facultative and strict anaerobes [38]. In this study, we included both types: *A. viscosus*, *S. oralis*, *V. parvula* and *P. gingivalis*.

In line with Vilarrasa et al. [35], who observed no significant difference in bacterial counts across titanium groups, our results showed that the metabolic activity of the multispecies biofilm remained unaffected by the presence of metal particles. However, a significant increase in the quantity and activity of *A. viscosus* and *V. parvula* was detected compared to the TCP control (*p* < 0.05), while *S. oralis* and *P. gingivalis* counts were significantly reduced (*p* < 0.05) (Figure 4 and Figure 5). This selective response could be related to the ability of *A. viscosus* and *V. parvula* to thrive in microenvironments created by metal particles, which favor bacterial adhesion and nutrient retention, together with the possible selective effect of metal ion release. Similarly, Violant et al. [37] identified *V. parvula* as the predominant species in a multispecies biofilm grown on titanium. These minor fluctuations in metabolic activity and LDH release at higher dilutions are not statistically significant and likely reflect biological variability and heterogeneous exposure conditions rather than a true inverse dose–response relationship.

Surface characteristics also influence fibroblast behavior. Fibroblasts spread more readily on smooth than on rough surfaces, and connective tissue adhesion is modulated by surface properties [13,30,46,47,48]. Beheshti et al. [30] demonstrated that surface roughness and chemical changes introduced during implantoplasty significantly influence fibroblast morphology, proliferation, and cytokine production. While such modifications promote fibroblast growth, rougher surfaces (e.g., SLA) may elicit stronger inflammatory responses, reflected in elevated IL-6 and MCP-3 levels. In agreement with our findings, fibroblasts on IP-treated implants showed no reduction in viability or metabolic activity (Figure 2 and Figure 3) and maintained an elongated morphology with ovoid nuclei and parallel alignment. By contrast, cells in the positive control group showed significantly impaired activity (*p* < 0.05).

The viability of SaOs-2 osteoblasts on IP-treated implants has also been validated. Toma et al. [31] found that despite significant alterations to surface morphology and wettability, IP-treated titanium still supported SaOs-2 proliferation and alkaline phosphatase (ALP), osteoprotegerin (OPG) and osteocalcin (OCN) production. These results are consistent with our own findings: IP alone did not impair osteoblastic viability or metabolic activity (Figure 2 and Figure 3).

In this study, the low cytotoxicity observed on IP-treated surfaces can be explained by a combination of factors. On the one hand, the resulting surface presents a smoother and more homogeneous topography that does not induce physical damage or mechanical stress to adherent cells. On the other hand, the release of metal ions (Ti, Al and V) from these surfaces was minimal and did not reach concentrations capable of altering cellular metabolism. This dual condition—absence of physical damage and low ion release—would explain why both fibroblasts and osteoblasts maintained high viability in the direct and indirect assays presented in Section 3.1 above, regardless of the dilution concentrations tested.

However, the aforementioned studies did not analyze the effect of the metal debris produced during implantoplasty. Moreover, the current controversy surrounding peri-implant diseases focuses on whether Ti particles are responsible for causing inflammation and osteolysis, as there is insufficient evidence correlating these conditions with metal debris [49]. Although the presence of particles in the peri-implant environment has been investigated, few studies have analyzed the titanium particles released during implantoplasty [1,26,27,32]. These particles generated during IP have been shown to cause a significant reduction in the viability of human fibroblasts [1,26,32] and SaOs-2 osteoblasts [26,32].

Several mechanisms responsible for the release of titanium particles and ions from dental implants have been described, including mechanical friction during implant insertion, surface wear generated by decontamination procedures, corrosion mediated by bacterial metabolites and inflammatory cells, and implantoplasty, which produces the greatest amount of metallic debris. Previous studies by our group [26] showed that Ti6Al4V surfaces and debris exhibit preferential release of vanadium ions compared with titanium or aluminum, a finding consistent with Barrak et al. [1], who reported significantly higher concentrations of V ions (0.12 ± 0.023 ppm) released from implantoplasty particles, with minimal release of Ti and Al. Although this evidence seems to indicate a potential biological relevance of vanadium, the present study did not evaluate ion release, and therefore could not establish any toxicity of the V ions.

Barrack et al. [1] studied particles released from grade Ti6Al4V titanium and observed the release of vanadium ions, which significantly reduced the viability of human gingival fibroblasts (HGFs) after 10 days of culture. In line with these findings, Toledano-Serrabona et al. [26] and Schwarz et al. [32] confirmed the cytotoxic effects of Ti particles from Ti6Al4V and pure titanium on fibroblasts and osteoblasts. Our results corroborate these findings and further suggest that bacterial contamination may amplify the cellular response to metal debris. Ti6Al4V particles contaminated with multispecies biofilm produced a pronounced cytotoxic effect, reducing fibroblast viability to below 25% and osteoblast viability to below 10% (Figure 8). This suggests that biofilm-derived metabolites may exacerbate the cellular response to metal debris, potentially by promoting surface corrosion, altering particle reactivity, or facilitating alloy degradation.

The immune response against metal particles varies according to particle size: large particles tend to be encapsulated, medium-sized particles are typically phagocytosed and eliminated by macrophages, and small particles may go unnoticed [50,51,52]. Implantoplasty residues are predominantly ultrafine (<100 nm) [11], a size range associated with increased cytotoxicity [53]. Ti6Al4V particles may also be more susceptible to corrosion and wear, potentially releasing vanadium and aluminum particles and ions with greater proinflammatory activity than cp Ti [54]. These particles can also activate inflammatory responses that increase cytokines such as IL-1β, IL-6, and TNF-α, promoting osteoclastogenesis and bone resorption and potentially compromising implant stability [36,55].

## 5. Conclusions

Titanium particles generated during implantoplasty significantly reduced fibroblast and osteoblast viability when contaminated with a multispecies biofilm, while implantoplasty-treated surfaces themselves remained cytocompatible. In addition, contaminated particles selectively promoted the growth of *Actinomyces viscosus* and *Veillonella parvula* without altering the overall biofilm metabolic activity. These findings emphasize that, although implantoplasty surfaces are biocompatible, the dissemination and contamination of metallic debris may pose a biological risk in peri-implant environments, underscoring the importance of minimizing particle release and contamination during clinical procedures.

## Data Availability

The original contributions presented in the study are included in the article. Further inquiries can be directed to the corresponding authors.

## Abbreviations

The following abbreviations are used in this manuscript:

IP	Implantoplasty
Ti	Titanium
Ti6Al4V	Titanium–Aluminum–Vanadium alloy
LDH	Lactate dehydrogenase
CFU	Colony-forming units
SEM	Scanning electron microscopy
EDS	Energy-dispersive X-ray spectroscopy
DMEM	Dulbecco’s Modified Eagle’s Medium
FBS	Fetal bovine serum
P/S	Penicillin-Streptomycin
TCP	Tissue culture plastic
BHI	Brain–heart infusion
ATCC	American Type Culture Collection
HFF-1	Human foreskin fibroblasts
SaOs-2	Osteoblast-like cells derived from human osteosarcoma
*S. oralis*	*Streptococcus oralis*
*A. viscosus*	*Actinomyces viscosus*
*V. parvula*	*Veillonella parvula*
*P. gingivalis*	*Porphyromonas gingivalis*
BET	Brunauer–Emmett–Teller theory
ISO	International Organization for Standardization
USA	United States of America
